# Mass spectrometry coupling of chip-based supercritical fluid chromatography enabled by make-up flow-assisted backpressure regulation

**DOI:** 10.1007/s00216-024-05381-y

**Published:** 2024-06-22

**Authors:** Chris Weise, Johannes Fischer, Detlev Belder

**Affiliations:** Institute of Analytical Chemistry, University of Leipzig, Linnéstrasse 3, 04103 Leipzig, Germany

**Keywords:** Chip chromatography, Supercritical fluid chromatography, Microfluidics, Mass spectrometry

## Abstract

This work introduces a novel microfluidic backpressure pressure control developed for chip-based supercritical fluid chromatography (chipSFC). The presented on-chip pressure control mechanism involves the post-column addition of a viscous make-up stream, which enables pressure regulation within the range of 73 to 130 bar range. In contrast to approaches using mechanical backpressure regulators, this chip-based make-up-assisted pressure regulation offers a wear-free alternative that functions entirely through fluidic means and contributes minimally to extra column volume. It prevents phase separation of the supercritical mobile phase and, therefore, expands the analytical scope of chipSFC to detection systems with an ambient pressure interface. This was demonstrated by a proof-of-principle experiment, where a model mixture was separated within 30 s and detected using atmospheric pressure ionisation mass spectrometry.

**Graphical abstract:**

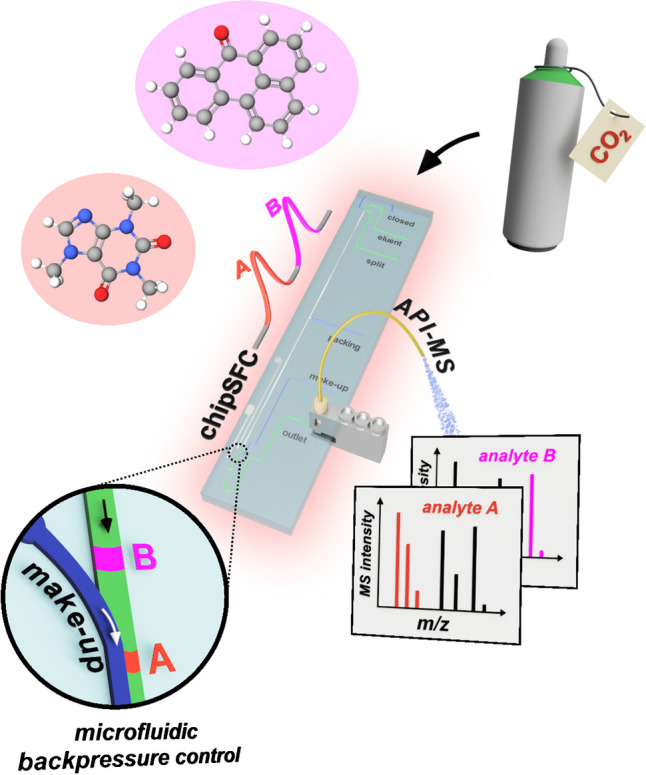

**Supplementary Information:**

The online version contains supplementary material available at 10.1007/s00216-024-05381-y.

## Introduction

One of the driving forces in instrumental analysis is the development of innovative instrumentation capable of generating high-quality data in the shortest possible time. This is necessary as time efficiency becomes increasingly important when dealing with large sample sizes in industry and research. In such high-throughput workflows, liquid chromatography-mass spectrometry (LC/MS) has proven to be an indispensable tool due to its selectivity and sensitivity, making it a prime target for speeding up runtime [1–3].

In addition to multi-parallel LC–MS approaches that enhance sample throughput without extending analysis time, strategies involving liquid mobile phases at high pressures (up to 1300 bar) or temperatures (up to 250 °C) are used to expedite individual analyses [4–9]. However, the extreme conditions of UHPLC and HTLC can lead to frictional heating and thermal mismatching, compromising separation efficiency [10–13]. Since the speed of chromatographic separations is significantly influenced by the eluent’s viscosity and diffusivity, using supercritical CO_2_ (scCO_2_) as a mobile phase shows potential for achieving high-speed chromatography.

In supercritical fluid chromatography (SFC), the unique properties of scCO_2_ can be utilised under relatively mild conditions (73.8 bar/31 °C) [14, 15]. Above these conditions, CO_2_ enables rapid mass transfer between mobile and stationary phases, increasing separation efficiencies even at high flow rates. Unlike other apolar liquids, scCO_2_ is miscible with polar modifiers, allowing he use of various stationary phases ranging from normal phase to reversed-phase materials [16, 17]. This versatility makes (SFC) attractive for both chiral and achiral analysis in the pharmaceutical and food industries [18–23].

Recently, the accelerating power of scCO_2_ in separation science has been further advanced with the introduction of miniaturised chip-based SFC (chipSFC) [24]. This approach combines the favorable kinetic properties of low-viscosity scCO_2_ with a functional glass chip’s small volume, enabling unprecedented chromatography speed [25–27]. Additionally, the adverse effects of extra column dispersion on separation efficiency are significantly minimised by seamlessly integrating individual chromatographic entities, such as injection, separation, and detection, on a single chip [28].

Although SFC has been successfully combined with various detection systems, coupling SFC with mass spectrometry poses a unique technical challenge. One primary concern is the risk of uncontrolled decompression of the supercritical eluent, which can rapidly convert into gaseous CO_2_ after leaving the column. This sudden change results in unstable signals, alterations in solubility, and reduced sensitivity [29]. To address this challenge, pressure regulation of the post-column region using conventional SFC backpressure regulators (BPRs) is a common approach. This is typically employed for SFC-UV or RI detection and has also been utilised in the first chip-based SFC approach using fluorescence detection [29–32]. Unlike optical spectroscopic methods, mass spectrometry detects analyte ions in the gas phase. This circumstance allows mass spectrometry to leverage the depressurisation process in chipSFC to achieve improved solvent desolvation and superior nebulisation before ionising a wide range of analytes [33, 34]. To utilise the expanding supercritical eluent for mass spectrometry, it is essential to have a compact pressure control device in the post-column region of the microchip. Conventional mechanical backpressure regulators are unsuitable for coupling chip-based SFC with MS due to their large internal volumes. Recently developed miniaturised BPRs address this challenge,yet adapting them for chip-based SFC MS remains an open challenge [35]. An intriguing alternative to mechanical BPR involves a hydraulic approach, where a high viscosity fluid is introduced to the effluent stream after the column. This configuration allows the dosing point pressure to be adjusted using the make-up flow pump [36–39].

**Fig. 1:**
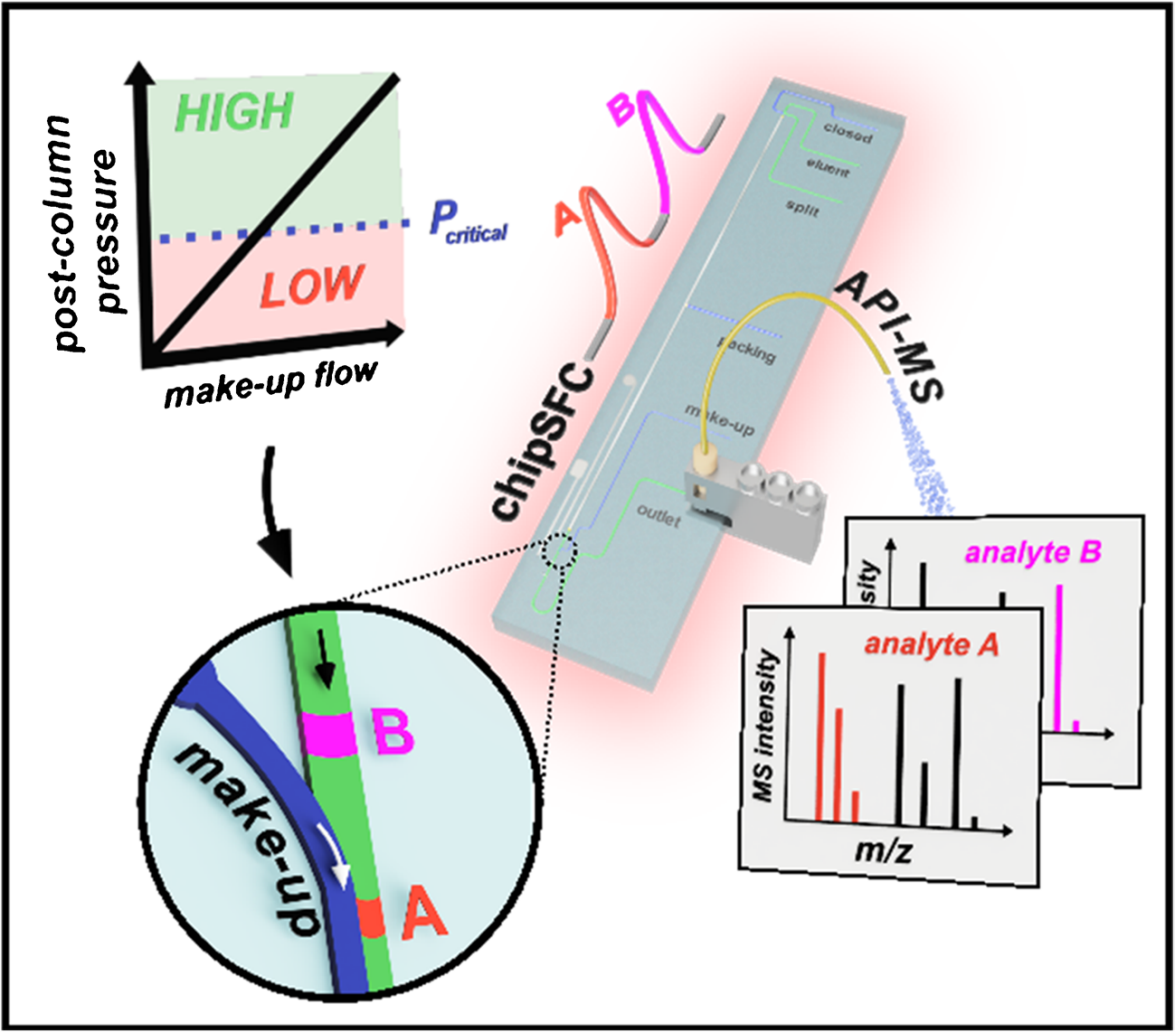
Conceptional overview of the MS coupling of chip-based supercritical fluid chromatography (chipSFC-MS). The approach presented utilizes the high viscosity of a make-up stream to control the post-column pressure on the chip. This prevents premature decompression of the supercritical effluent and enables the detection of rapid chromatography by atmospheric pressure ionization mass spectrometry (API-MS)

This study aims to overcome the obstacles associated with coupling chipSFC with mass spectrometry by integrating a make-up flow-assisted principle for the post-column pressure control on the chip (Fig. 1). Pressure measurements and microscopic inspections were conducted at the confluence point to assess the impact of the make-up flow on the decompression of the CO_2_-based eluent in the developed chipSFC system. The effectiveness of this unique chipSFC-MS interfacing approach was evaluated through rapid separations using a model mixture.

## Materials and methods

### Chemicals and reagents

Solvents used, such as methanol, ethanol, isopropanol, and acetonitrile, were purchased in gradient grade purity (≥ 99.9%) from VWR (USA). Additionally, formic acid (99%, Sigma) and purified water from smart2pure (TKA, Germany) were used. Pressurised CO_2_ (purity grade N45) from Air Liquide (France) was used with different modifier compositions as supercritical eluent. Chemicals for chip functionalisation include H_2_SO_4_ (37%), acetic acid (98%), and 3-(trimethoxysilyl)propyl methacrylate (98%), 2,2-dimethoxy-2-phenylacetophenone (99%), 1,3-butanediol diacrylate (98%), and butyl acrylate (99%) all bought from Sigma-Aldrich (Germany). 4-Amino-7-methylcoumarin, abbreviated as c120 (purity ≥ 99%), fluoranthene (purity 99%), and benzo[k]fluoranthene (purity 98%) from Sigma-Aldrich (Germany), were used as fluorescent samples for injection evaluation. Benzanthrone (purity ≥ 98%, Sigma-Aldrich) and c120 were the test sample mixture for chipSFC MS experiments. Sample stock solutions were filtered by a 0.22-µm pore size Teflon filter cartridge and diluted to the desired concentration. For achiral separations, XBridge C18-BEH particles (dp = 2.5 µm, Waters, USA) were used as the stationary phase of the chip-based microcolumn. Fused silica capillaries (OD 360 µm) coated with polyimide (Molex, USA) of various diameters (ID 5, 10, and 20 μm) were used as a restrictive emitter. All other capillaries were sourced from IDEX (USA). Static and dynamic backpressure regulators were acquired from IDEX (USA) and VICI (Switzerland). The heating cartridge for the BPR was purchased from Selerity Technologies, USA.

### Microchip layout

**Fig. 2:**
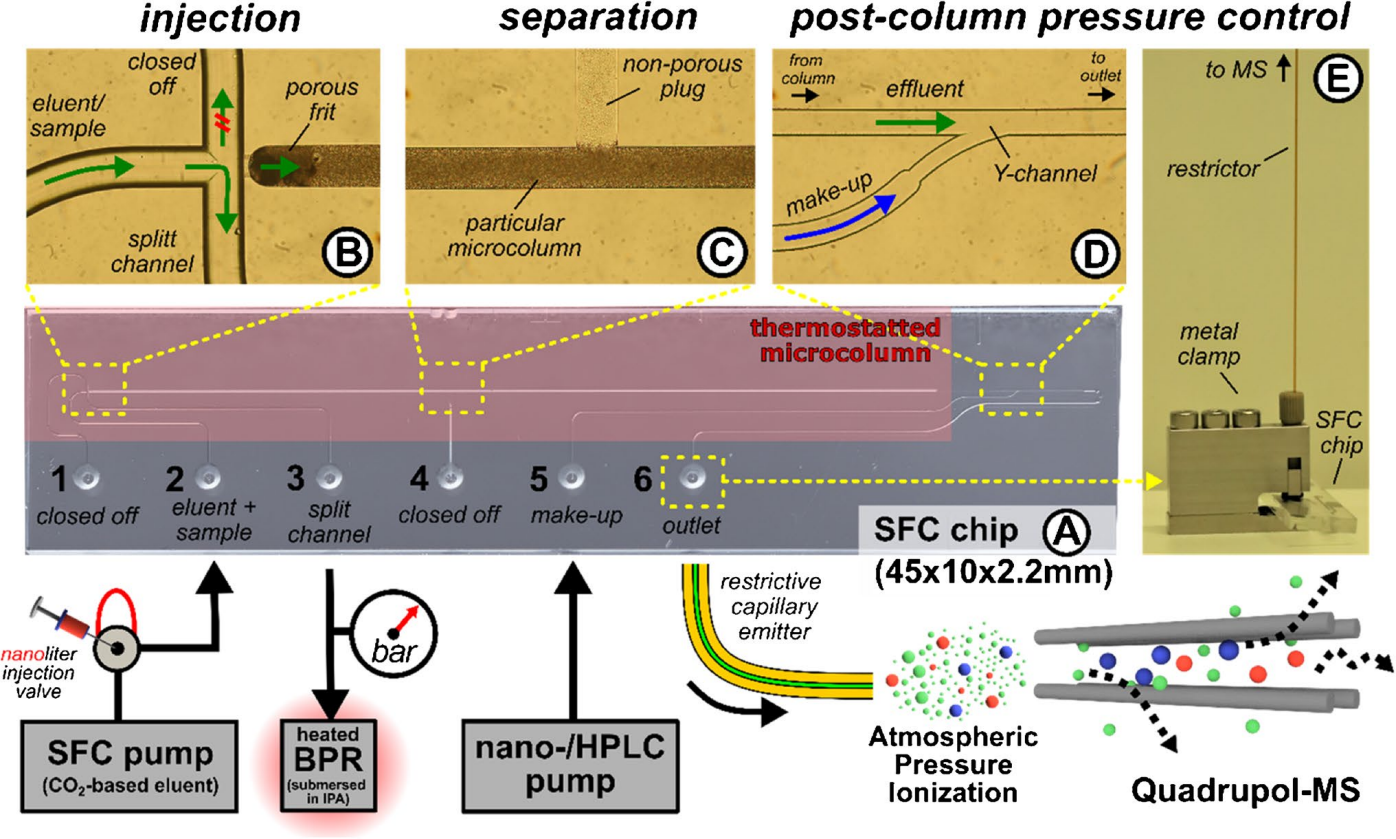
Instrumental setup for the operation of chipSFC MS. The schematic shows **A** the functionalized SFC microchip and the external pumping and backpressure stabilization peripherals connected to it. Microscopic insights into the structural features and their fluidics that are relevant to the **B** injection, **C** separation, and **D** post-column pressure control are shown. **E** Side view of the off-chip implementation of a restrictive capillary emitter using a pressure stable metal clamp. The setup is placed in front of a single quadrupole MS for mass analysis. Ionization occurs under ambient conditions. More details about the setup can be found in Fig. S4

The instrumental setup, illustrated in Fig. 2, features the borosilicate (BOROFLOAT®33) microfluidic chip (45 mm $\times$ 10 mm $\times$ 2.2 mm) designed and manufactured by iXfactory (now part of Micronit, Germany), following our specifications as described in previous publications on chip-based chromatography [24, 32, 40]. The microchip (Fig. 2A) includes microfluidic channels, which the fluidic periphery can access via six conical-shaped openings integrated into the top plate. The bottom part of the microchip, prepared through photolithography and HF-etching, features a microfluidic cross for sample injection (Fig. 2B), a column compartment (35 mm length, 90 µm width, 45 µm depth, Fig. 2C), and a post-column Y-junction (Fig. 2D).

The column compartment was prepared in four sequential steps. Initially, microchannels were cleaned and treated with silane-based methacrylate [27]. Acrylate-based porous frits were inserted at both ends to retain particles (Fig. 2B). C18 BEH particles (1–3 mg/mL) were slurry-packed through the lateral channel in the center of the microcolumn. Finally, a pressure-tight seal was introduced by placing a non-porous, acrylate-based polymer plug into the lateral access channel (Fig. 2C). The precise insertion of both frits and the plug was performed using photopolymerization. The photo-polymerisation setup was equipped with a pinhole aperture to ensure a small spot size of the LED beam. In the post-column region of the microchip, the effluent channel merges with a channel for make-up addition in a Y-shaped structure (Fig. 2D), leading to the outlet port. Subsequently, the effluent flows to the outlet port into the off-chip MS interface, passing through a channel with changing dimensions. A fused silica capillary (OD 360 μm, ID 10 µm, or 20 µm) acts as the restrictive emitter and is connected to the microchip’s outlet port via a metal clamp (Fig. 2E) [25]. The supplementary information provides additional details on cleaning procedures, surface functionalization, and the introduction of UV-cured polymeric structures.

## Method

### Microchip operation

The developed SFC microchip was connected to fluidic peripherals using pressure-stable clamps and PEEK capillaries to ensure operation. The corresponding instrumental setup is illustrated in Fig. 2. Therein, an SFC pump (Infinity II 1260 SFC, Agilent Technologies, USA) was employed to deliver the CO_2_-based eluent necessary for the separation process. Downstream of the SFC pump, an external nano volume valve (bore size 100 µm, VICI, Switzerland) was utilised to inject a 5 nL sample plug into the eluent stream. The nano volume valve was connected to the SFC microchip via a PEEK capillary (OD 360 μm, ID 50 μm, length 15 cm), allowing the sample plug to access the chip-based microcolumn for separation. The study employed two injection concepts with distinct strategies for transferring the sample from the nano volume valve to the column head. The sample flows directly onto the chip-based column in splitless injection concept.

In contrast, the split injection concept involves discarding a fraction of the sample before it enters the column. This is achieved by introducing a backpressure-stabilised split channel. The back pressure was maintained by a series of heated (Selerity Technologies, USA) and unheated backpressure regulators (IDEX, USA) submerged in isopropanol. A pressure gauge (Duratec, Germany) was implemented at the split channel for pressure metering. Depending on the flow rate required, either an HPLC pump (1260 infinity, Agilent Technologies, USA) or a nanoflow pump (Ultimate NCS-3500RS, Thermo Fischer Scientific, USA) was used to deliver a make-up stream in the post-column section of the chip. The SFC microchip was kept at 60 °C during operation using a custom microchip thermostat [32]. Before each measurement, the setup was equilibrated in isocratic mode with the desired make-up flow. Valve switching and simultaneous MS acquisition triggering were done automatically using Clarity (Data Apex, Czech Republic). Fig. S1 and Fig. S4 in the supplementary information provide more details about the instrumental setup and fluidic circuitry used.

### Fluorescence detection

An inverted microscope (IX-71, Zeiss, Germany) equipped with a mercury-vapor lamp (HBO 103 W/2, Osram, Germany) and a filter cube composed of an excitation filter (band-pass 350/50 nm), a beam splitter (dichroic mirror 380 nm), and emission filter (long-pass 390 nm) were used for fluorescence detection. The emitted photons were detected by a photo-multiplier (Hamamatsu Photonics) using an amplification voltage of 700 mV. The analog output of the PMT was digitalized and recorded by Clarity (Data Apex, Czech Republic). For fluorescence measurements, backpressure control was performed using conventional static BPRs and a single dynamic BPR connected to the chip outlet. A detailed version of the chipSFC fluorescence setup and the acquired data can be found in the supplementary information Fig. S1–S3.

### Mass spectrometric detection

The chipSFC-thermostat assembly was placed in front of the MS aperture, which was modified by removing the spray shield and the capillary cap before the measurement. Positive ion mode single quadrupole MS (6150B, Agilent Technologies, USA) was employed to detect the ion chromatograms of the protonated analyte ions within a mass range of m/z150–400 at a cycle time of 500 ms (2 Hz). The chipSFC MS setup details can be seen in supplementary information Fig. S4.

## Results and discussion

Figure 1 illustrates the goal of this study, which is to couple chip-based SFC with mass spectrometry to achieve fast and efficient chip chromatography using supercritical eluents. This represents a major challenge because transferring the CO_2_-based effluent to ambient conditions requires post-column pressure control to avoid premature gas formation. Previous chip-based SFC studies have used mechanical BPRs downstream of the detection to stabilize the post-column pressure [30]. Such an approach is unsuitable for MS coupling due to the large BPR volume the effluent must pass through to reach the MS emitter. Instead, incorporating a make-up fluid offers a promising strategy to regulate post-column pressure in a novel chipSFC MS interface.

### Split on-chip sample injection

The proposed chipSFC-MS interface was developed using a simplified split injection approach. This modification enabled more robust operation with a simpler fluidic setup compared to previous chipSFC studies [24]. The operational principle of the newly introduced split injection is based on dividing the sample plug into two fractions, discarding one while loading the other onto the column.

The microfluidic cross had to be converted to a tee-junction to implement this in the current chip layout. Therefore, one channel of the microfluidic cross (Fig. 2B) was sealed with a metal clamp and a capped PEEK capillary. The remaining channel served as a split channel and was backpressure-stabilized to maintain a constant pre-column pressure of 1–2 bar below the SFC pump pressure.

Since the flow in SFC is driven by pressure differences within the fluidic system, the proposed measure created a pressure drop necessary to accelerate the transfer of the sample plug in the pre-column section of the microchip.

**Fig. 3:**
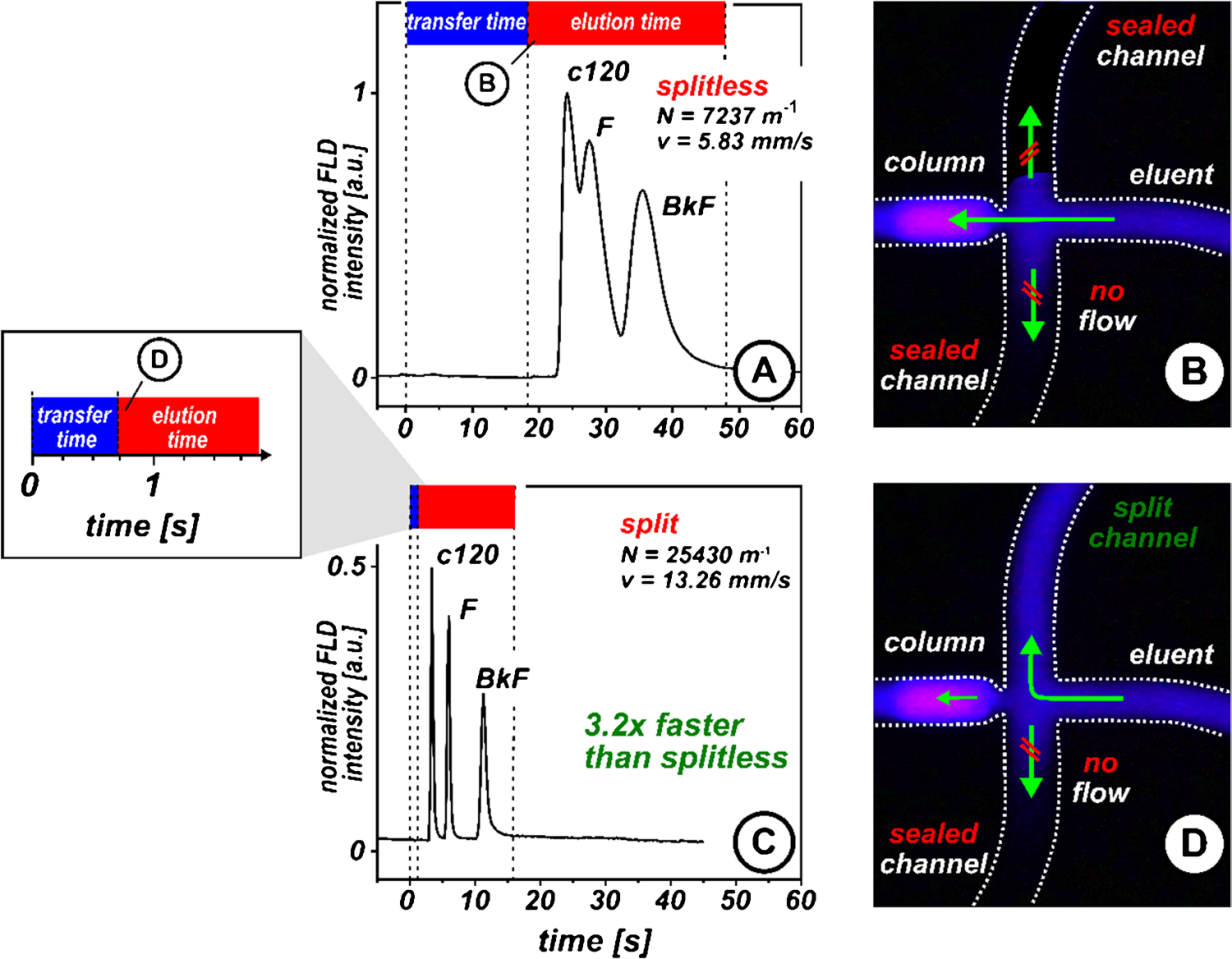
Comparison between splitless and split injection mode for chipSFC. The advantages of the split injection over splitless are illustrated by the normalized chromatograms obtained with chipSFC fluorescence detection using **A** splitless and **C** split injection. The transparent glass chip offers insights into the fluidics involved during **B** splitless and **D** split injection modes at the column head using a fluorescent analyte plug (c120, c = 500 µM dissolved in MeOH). By observing the column head, the transfer time (blue) and elution time (red) were determined and represented above each chromatogram. Transfer time refers to the time the sample plug remains in the precolumn volume, while elution time refers to the sample spends on the column. Experimental parameters of the acquired chromatograms are listed in the following: pre-column pressure, 160 bar; post-column pressure, 120 bar; stationary phase, C18 BEH, dp = 2.5 µm; column length, 35 mm; column temperature, 60 °C; eluent, CO_2_/MeOH 80:20 (v/v). A sample mixture of 7-amino-4-methyl-coumarin (c120, c = 400 μmol/L), fluoranthene (F, c = 1.90 mmol/L), and benzo[k]fluoranthene (B*k*F, c = 200 μmol/L) dissolved in MeOH. Linear velocity was calculated based on c120. Normalization was performed using the intensity data obtained by the splitless injection. Determination of theoretical plates is based on B*k*F. The green arrows indicate flow directions. Details about the fluorescence setup and the preliminary measurement made used can be found in the Fig. S1–S3

The split injection mode was compared with a splitless configuration by separating a mixture of three polycyclic aromatic hydrocarbons using a chipSFC fluorescence setup to test this hypothesis. The resulting chromatograms in Fig. 3A and C demonstrate that split injection outperforms splitless injection, showing improvements in key chromatographic parameters, including linear velocity (5.83 mm/s for splitless mode, 13.26 mm/s for split mode) and the number of theoretical plates (7237 m^−1^ for splitless mode, 25,430 m^−1^ for split mode).

Since the mobile phase velocity on the column remains unchanged regardless of the injection mode, it was suspected that the observed performance differences between the two injection modes were due to an insufficient pressure drop caused by the low viscosity of the subcritical fluid in the pre-column section.

In the splitless mode, the pre-column pressure drop primarily depends on the friction between the eluent and the tubing wall [41]. Given the minimal friction, the pre-column pressure drop and the flow velocity are likewise minimal. Consequently, this injection strategy is not recommendable.

In split injection mode, this drawback is circumvented by a split channel that vents the pre-column section to atmospheric pressure. The dynamic BPR, which is connected to the split channel, allows the pressure drop and, thus, the flow velocity in the pre-column area to be adjusted.

To investigate this further, we examined the chronological order of processes at the column head using a fluorescent sample plug. Figure 3B shows the corresponding microscopic images of the sample plug at the column head in splitless injection mode, and Fig. 3D shows split injection mode. In this context, the transfer time was determined, which refers to the time the sample plug to moves from the external injection valve to the chip-based column head. Interestingly, the on-chip split injection exhibited a 25-fold faster transfer time (720 ms) than the splitless injection (18 s). These results confirm the hypothesis of insufficient pressure drop in the pre-column section when only relying on the column as the flow restrictor. The backpressure-stabilised split channel resolves this issue by creating the necessary pressure drop for rapid sample transport to the column head.

Based on the observations of the fluorescent sample plug at the column head, it was evident that only a fraction of the sample was loaded onto the column (Fig. 3D). This circumstance was reflected in the heights of detected chromatographic peaks. In split mode, only 68.5 to 70% of the peak heights detected in splitless mode were achieved. This indicates that the performance improvements observed were not entirely due to increased flow velocities in the pre-column section but also benefited from the reduced sample load.

To further investigate this, the split ratio at the tee junction was determined. Therefore, the areas of all detected peaks in split injection mode were summed and normalised relative to results gained during splitless injection. The results indicated that only about 7.2% of the peak area was detected in the split mode. This suggests that about 360 pL of the original 5 nL sample plug volume was injected into the column in split injection mode.

Injecting a sample plug of this volume encounters the existing column dimensions, as it avoids overloading the column capacity and thus contributes to a more efficient separation.

### Post-column make-up flow addition

After the on-chip implementation and evaluation of the split mode injection, the focus shifted towards integrating the make-up-assisted pressure regulation in the post-column region of the SFC microchip.

During a make-up-assisted pressure regulation, a viscous and incompressible make-up liquid is added to the supercritical effluent to adjust the post-column pressure. This fluidic element requires a post-column junction for make-up dosing and a restrictive outlet channel.

 The make-up flow is added via a Y-branch behind the column using a nanopump with the presented chip layout. In addition, a preliminary test was carried out to determine whether the existing post-column microchannel provides enough restriction for the proposed make-up-assisted pressure regulation. Therefore, the mechanical backpressure regulator used during the fluorescence measurements was disconnected from the chip’s outlet port to simulate a restrictive outlet channel. Unexpectedly, this post-column configuration led to immediate eluent outgassing even before make-up addition, indicating that the dimensions of the post-column channel were not restrictive enough to prevent phase separation of the CO_2_-based mobile phase.

To increase the restriction of the post-column channel, a narrow-diameter fused silica capillary was connected to the outlet port at the back of the SFC microchip using a custom-made metal clamp [25]. Using a fused silica capillary restrictor was convenient because it allows flexible restriction adjustment, either by reducing the capillary length or replacing the entire capillary restrictor.

During this experimental phase, capillaries of different dimensions were connected to the SFC chip to evaluate their backpressure for an 80:20 v/v CO_2_/MeOH mobile phase at 160 bar and a 90:10 v/v MeOH:H_2_O, 0.1% FA make-up flow. The results of these experiments are displayed in Fig. S5. Under the selected conditions, all tested capillary restrictors prevented phase separation, although the flow rate and viscosity of the make-up stream influenced their backpressures. Smaller restrictor dimensions (IDs of 5 or 10 µm) enabled lower nanoliter-per-minute make-up flow rates. In comparison, larger capillary IDs (ID of 20 µm) accommodated microliter-per-minute make-up flow rates, exhibiting a wider adjustable pressure range and reduced susceptibility to clogging.

**Fig. 4:**
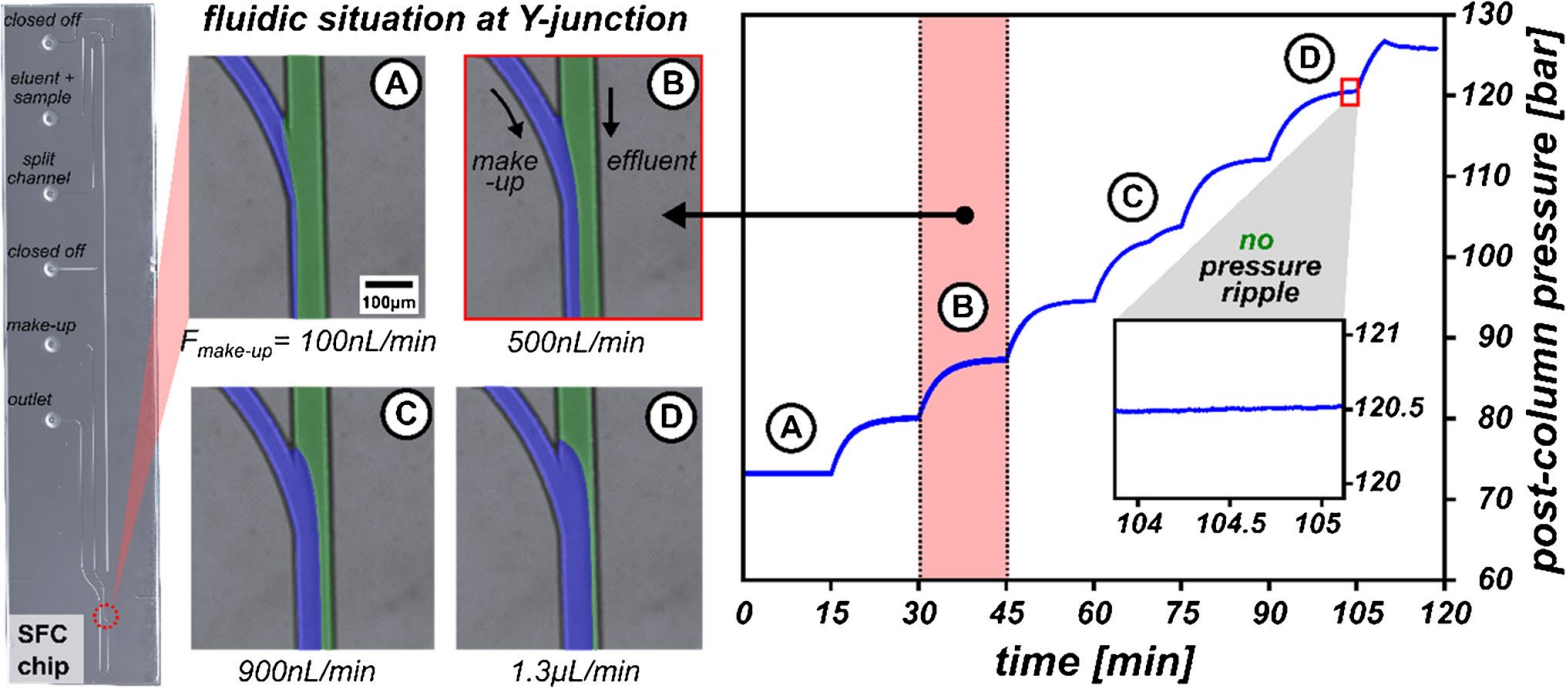
Working principle of the post-column backpressure regulation for chipSFC MS. (left side) Photographic inspection of the post-column Y-junction during make-up flow addition. The confluence of effluent (green) and make-up liquid (blue) at different make-up flow rates (**A–D**) and (right side) its corresponding post-column backpressure curves. The insight shows the absence of the pressure ripple, illustrating the pressure damping effect and the accuracy of the make-up-assisted control mechanism. Post-column pressure was monitored using the pressure values of the make-up pump. Experimental parameters are listed: pre-column pressure, 160 bar; column temperature, room temperature; eluent, CO_2_/MeOH 80:20 (v/v); make-up composition, MeOH/H_2_O 90:10 (v/v); restrictor dimensions, fused silica capillary with OD 360 μm, ID 10 μm, and length of 20 cm

To demonstrate the working principle of a microfluidic make-up-assisted backpressure control on the SFC microchip, a restrictor with an inner diameter of 10 µm and a capillary length of 20 cm was selected. The corresponding microscopic inspections in Fig. 4 illustrate the fluidic situation at the dosing point, where both the viscous make-up stream and the subcritical mobile phase exhibit a laminar flow regime. Increasing the make-up flow rate compresses the effluent, as evidenced by the shift in the phase boundary. Due to its compressibility, the effluent balances out pressure fluctuations, highlighted by the absence of the pressure ripple under high-pressure conditions. Using the selected restriction capillary, post-column pressures can be adjusted from 73 to 123 bars with a make-up composition of 90:10 v/v MeOH: H_2_O, 0.1% FA at a flow rate ranging from 0.1 to 1.5 µL/min.

**Fig. 5:**
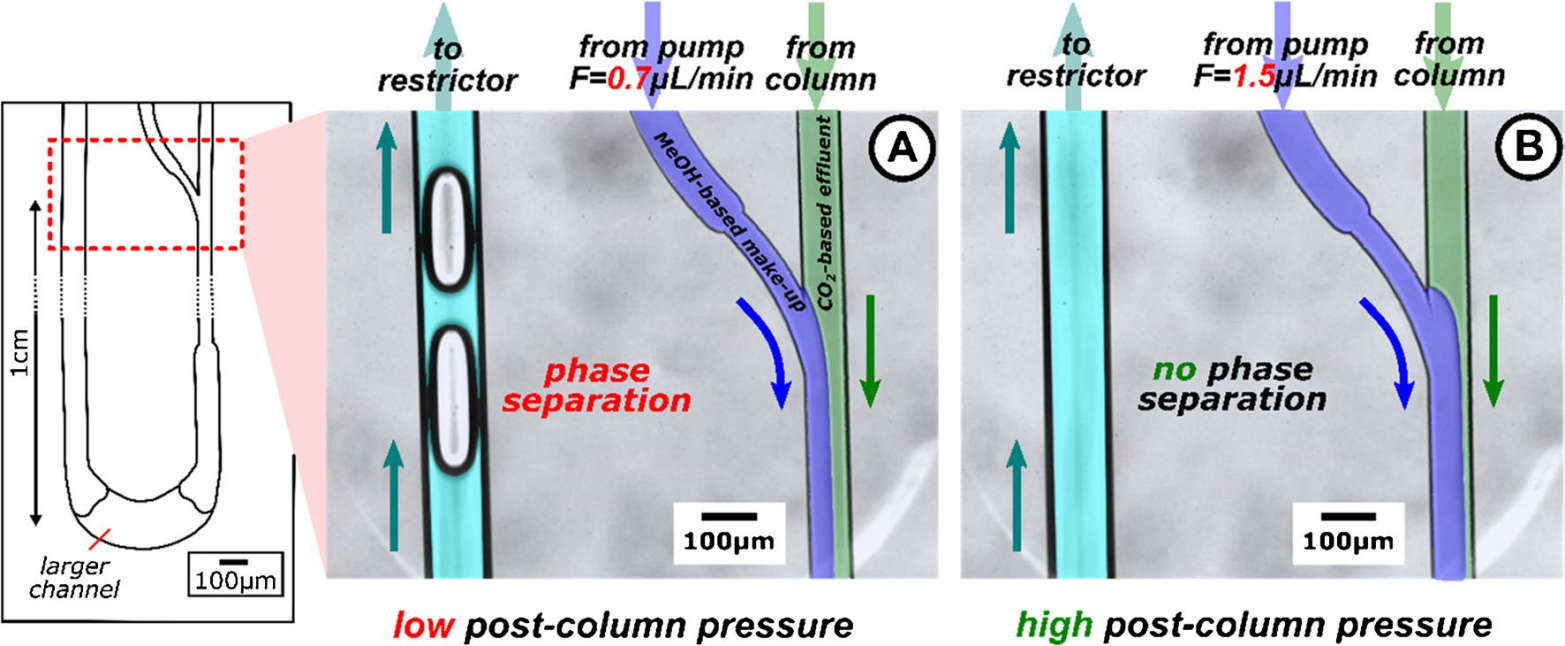
Impact of the make-up-assisted backpressure regulation on phase behavior in the post-column section of the SFC microchip. The schematic on the left provides an overview of the post-column region of the employed microchip. In there, the red dotted box highlights the fluidic situations at the Y-junction and the downstream channel under different pressure conditions. In (**A**), a low post-column pressure (93 bar) is created by a make-up flow rate of 0.7µL/min. (**B**) shows that higher make-up flow rates (1.5 µL/min, post-column pressure: 123 bar) can prevent phase separations of the liquefied CO_2_ effluent. For clarity, the effluent (green) and make-up streams (blue) are labeled, with the turquoise color representing the mixture of both fluidic streams. The observed mixing is caused by the channel enlargement downstream of the Y-junction. Experimental conditions are listed as follows: 160 bar pre-column pressure; column temperature, room temperature; eluent, CO_2_/MeOH 80:20 (v/v); make-up composition MeOH/H_2_O 90:10 (v/v); restrictor dimensions, capillary length 20 cm and inner diameter 10 μm. More details about origin of the phase separation are provided in Fig. S8

The importance of make-up-assisted backpressure regulation for effective post-column transfer is highlighted in Fig. 5. This figure shows a microscopic image of two sections of the post-column channel under different post-column pressure conditions. Under both post-column pressure conditions, two parallel flow streams are observed at the Y-channel junction. At a post-column pressure of 123 bar (Fig. 5B), the outgoing channel exhibited a homogenous effluent, suggesting a mixing between the effluent and the make-up stream. In contrast, gas bubbles were evident at a post-column pressure of 93 bar (Fig. 5A). This indicates that a post-column pressure above 93 bar is required to avoid phase separation of the CO_2_-based effluent. This was surprising because, at room temperature, the CO_2_ boiling pressure within such a binary (CO_2_-MeOH) mixture remains below the critical pressure. Therefore, entering the two-phase region of the specific vapor–liquid equilibrium seems unrealistic [34].

Microscopic examination after the Y-branch revealed that the rapid pressure drop caused by channel enlargement is responsible for mixing the effluent and make-up stream, as well as gas bubble formation (Fig. S8). The transition from post-column channel A (with 60 μm, depth 10 μm) to channel B (width 90 μm, depth 40 μm) forces the effluent to expand and reduce its density and velocity. These rapid changes create shear forces that induce eddies and vortices responsible for disrupting the laminar flow. To resolve this, future chip designs should ensure laminar flow by maintaining restrictive channel dimensions like those in post-column channel A. Additionally, using an in-line, multi-path restrictor, such as a porous frit, can create the needed pressure drops without adding extra column volume [42, 43].

### Model separation using chipSFC MS

With the working make-up-assisted backpressure regulation in place, the chromatographic performance of the developed system could be tested in a proof-of-principle experiment.

Therefore, a make-up composition of methanol and water (90:10 v/v) was chosen. Adding water increased the make-up liquid’s viscosity, enabling a lower flow rate during operation. Since both analytes dissolve in methanol, it prevents analyte precipitation during CO_2_ decompression. Formic acid was added to the make-up to enhance potential ionisation [38].

**Fig. 6:**
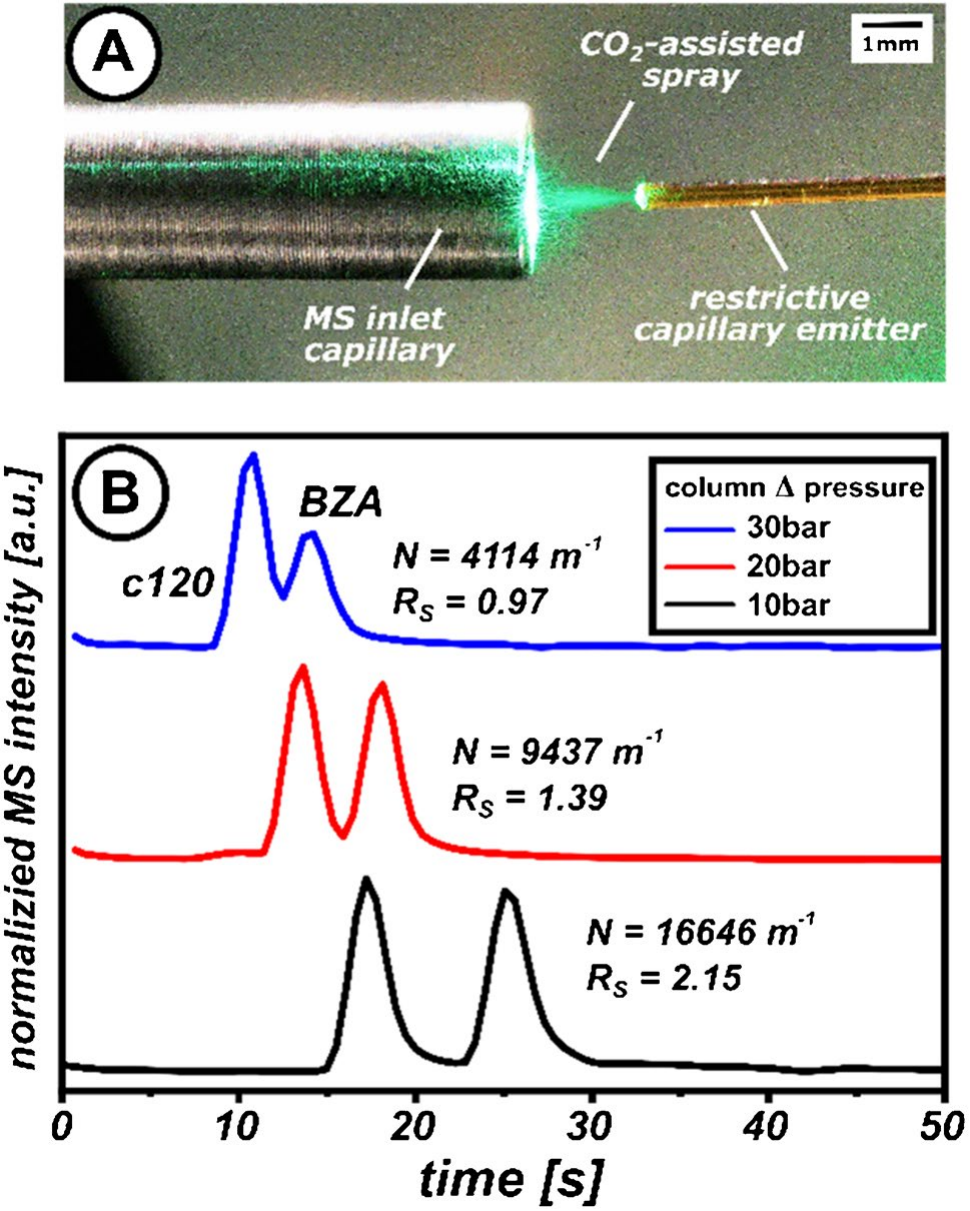
Proof-of-principle experiment demonstrating chipSFC MS with make-up-assisted backpressure regulation. **A** Photographic image of the MS inlet during the expansion of the CO_2_-based effluent. A green laser pointer illuminated the expanding spray plume. **B** Ion chromatogram of a model mixture separated using chipSFC MS at different column pressure drops. Experimental parameters are listed as follows: pre-column pressure, 160, 150, and 140 bar; column temperature 60 °C. Eluent, CO_2_/MeOH 80:20 (v/v); stationary phase, C18 BEH, 2.5 µm; column length, 35 mm; post-column pressure, 130 bar; make-up composition MeOH/H_2_O 90:10 (v/v), 0.1%FA. Restrictor: 20 μm ID, 80 cm length, make-up flow rate 5 μL/min. Sample mixture: c120 and BZA (each 1 mmol/L) dissolved in MeOH. MS parameter: single quadrupole MS, positive ion mode, mass range m/z 150–400. Cycle time: 500 ms, capillary voltage 4.5 kV, dry gas temperature 350 °C, fragmentor voltage 200 V (c120) and 230 V (BZA), Theoretical plates are calculated based on the BZA

The developed SFC microchip assembly was positioned in front of a quadrupole MS. The outlet tip of the restrictor, serving as an emitter, was placed 2 mm in front of an MS inlet capillary at high electrical potential (Fig. 6A). Under the given conditions, a non-pulsating CO_2_-assisted spray was generated between the emitter tip and the MS inlet capillary, producing a stable TIC signal of approximately 4.5 × 10^6^ counts (Fig. S6). We further assessed the system’s suitability by injecting 7-amino-4-methylcoumarin (C120) and utilising the quadrupole’s selected ion monitoring (SIM) capability to enhance sensitivity by excluding non-informative background ions. The resulting SIM chromatograms of protonated molecular ions are depicted in Fig. S6. The unsegmented signal peak, reaching a maximum height of 2.3 × 10^6^ counts, confirms the occurrence of post-column transfer, with decompression having a minimal effect on the chromatogram’s integrity.

Since retention in SFC is sensitive to pressure and temperature, we tested their impact by separating a two-compound mixture of c120 and benzanthrone (BZA). Adjusting the column pressure drop to 10 bar enabled baseline separation of both compounds within 30 s (Fig. 6B). In contrast, separations at different temperatures caused only minor differences in retention time (Fig. S7), indicating that temperature has a less pronounced effect compared to column pressure drop or modifier content.

## Conclusion

This proof-of-principle study is the first to demonstrate chipSFC coupled with ambient ionisation mass spectrometry. The microfluidic glass chip uses a backpressure-stabilised split channel to overcome low pre-column pressure drop, achieving picoliter sample plugs suitable for packed chip-based columns. The post-column integration of a make-up stream enables on-chip pressure regulation up to 130 bar without pressure ripple, allowing high-speed separation of a model mixture in under 30 s. These results highlight the potential of chipSFC with ambient detection systems. Future improvements, such as employing smaller stationary phase particles and further reducing the post-column volume, could lead to even more efficient separations in SFC microchips.

### Supplementary Information

Below is the link to the electronic supplementary material.

Supplementary file1 (DOCX 6.02 MB)
