# Supplementary material for: Mass spectrometry coupling of chip-based supercritical fluid chromatography enabled by make-up flow-assisted backpressure regulation
Source: Anal Bioanal Chem. 2024 Jun 22;416(20):4447–56. doi: 10.1007/s00216-024-05381-y (PMC11294422; doi:10.1007/s00216-024-05381-y)
Supplement: Supplementary file 1 — Supplementary file1 (DOCX 6.02 MB) [file 216_2024_5381_MOESM1_ESM.docx]

Supporting information

Mass spectrometry coupling of chip-based supercritical fluid chromatography enabled by make-up flow-assisted backpressure regulation

Chris Weise^a^, Johannes Fischer^a^ and Detlev Belder^a^*

# ^a^ Institute of Analytical Chemistry, University Leipzig, Linnéstrasse 3, 04103 Leipzig, Germany

## Corresponding author:

*E-mail Detlev Belder: belder@uni-leipzig.de

## **Table of content**

***Functionalization of the microchip***

Chip cleaning, Surface modification, and insertion of UV-sensitive polymers page - S2

***Fluorescence experiments***

Figure S1 – Experimental setup for chipSFC fluorescence page – S3

Figure S2 – Impact of mobile phase modifier page – S4

Figure S3 – Impact of column pressure drop page – S5

***Mass Spectrometry experiments***

Figure S4 – Experimental setup chipSFC Mass Spectrometry setup page – S6

Figure S5 – Evaluation of different restrictor dimensions page – S8

Figure S6 – Ion chromatograms acquires by chipSFC MS page – S9

Figure S7 – Impact of temperature page – S10

***Additional observation***

Figure S8 – Observation of the post-column section page – S11

References page – S12

## The following section details the functionalization of the microchip, beginning with a cleaning procedure to remove organic and inorganic residues remaining in the microfluidic channels after etching. This thorough cleaning is essential to prepare the channels for efficient chemical modification. The procedures described here are based on previously published protocols [1–3].

## Cleaning and surface modification of microchip

The chip channels underwent a series of rinsing steps to clean the microchannel network. First, they were flushed with high-purity water followed by methanol. Subsequently, the channels were incubated in concentrated sulfuric acid (H_2_SO_4_) for 20 minutes. After discarding the sulfuric acid and performing rinses with aqueous and methanolic solutions, the microchip was left to incubate overnight in a silane-based methacrylate solution for surface modification.

The methacrylate solution was composed of ethanol, acetic acid, and 3-(trimethoxysilyl)propyl methacrylate in a ratio of 4:3:3. Following surface modification, the chip was stored in a casting solvent containing 60% v/v  acetonitrile, 20% v/v ethanol, and 20% v/v 50 mM aqueous phosphate buffer at pH 6.8 before undergoing further functionalization.

## On-chip insertion of UV-sensitive polymeric frit and plug structures

The pre-polymer solution used to create non-porous plugs and porous frits contained 2,2-dimethoxy-2-phenylacetophenone (7 mg for porous frits, 6 mg for non-porous plugs) as a photo-initiator along with different acrylic monomers.

For the porous frit structure, the solution included 1,3-butanediol diacrylate (165 μL), butyl acrylate (160 μL), and a casting solvent (670 μL). In contrast, the pre-polymer solution for the non-porous plug structure consisted of 1,3-butanediol diacrylate (877.5 μL) and methanol (117.5 μL) only. Each pre-polymer solution also contained 5 μL of 3-(trimethoxysilyl)propyl methacrylate to facilitate the bonding of the polymer structure with the modified surface of the channel walls.

The microchip was filled with the corresponding pre-polymer solution to generate the polymeric frits or plugs by photo-polymerization. Using an inverse microscope (IX-71, Zeiss, Germany) equipped with a 355nm LED (Thorlabs, Germany) and 40x objective (Olympus, Germany), the frit structure was created at desired locations within the microchip. An additional pinhole aperture was used to ensure a small spot size of the LED beam. Porous frits were inserted before introducing the particle slurry, while non-porous plugs were inserted after packing the slurry to seal the column tightly. After successful insertion, methanol flushed any remaining pre-polymer solution off the microchip.

## Fig. S1 – **chipSFC** fluorescence setup

##
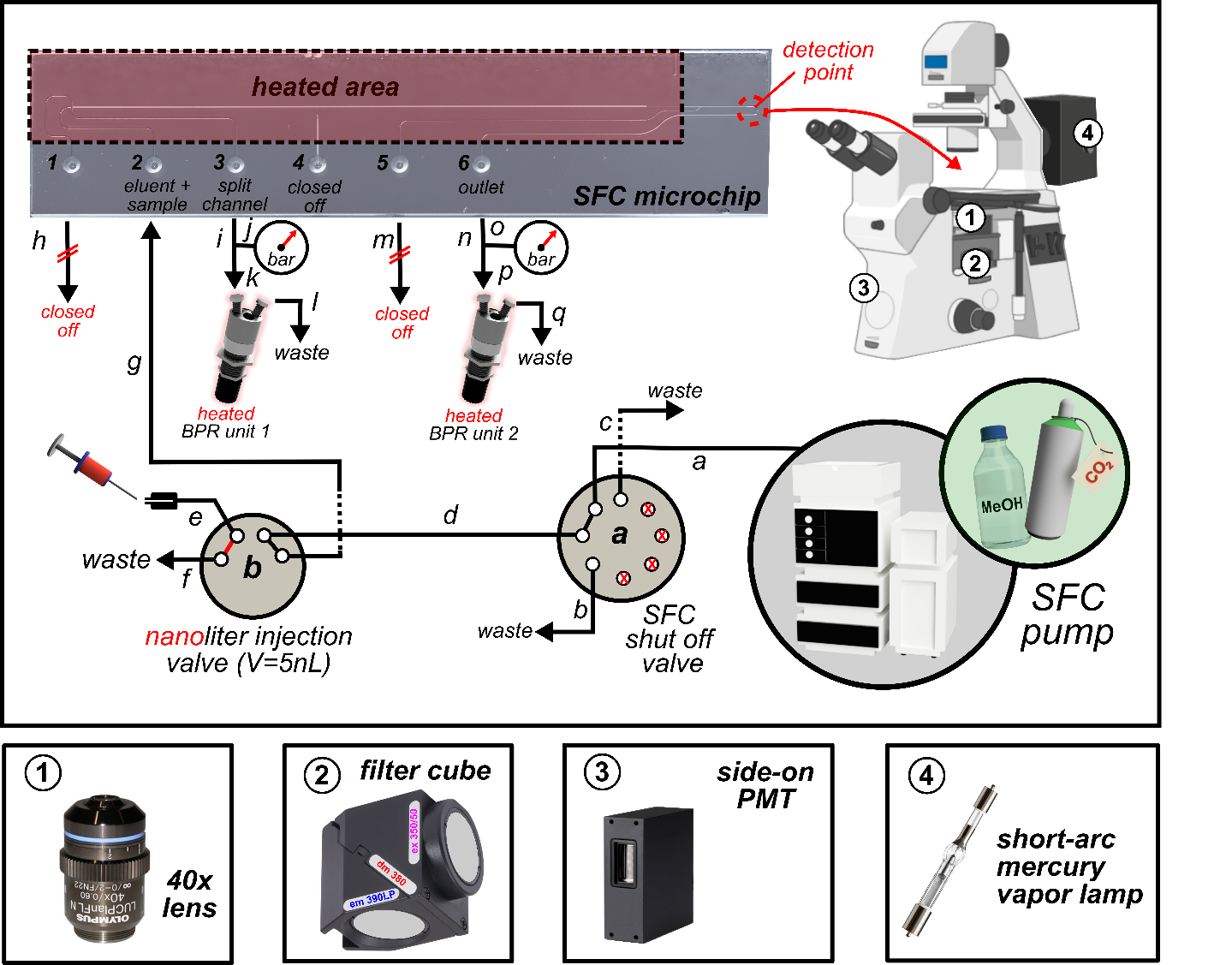


###

### **Fig. S1** chipSFC fluorescence setup used to evaluate split and splitless injection. The schematic displays the split injection mode. For splitless operation, the BPR unit 1 was removed, and the split channel was closed off. The fluidic circuitry is driven by an SFC pump (1260 Infinity SFC, Agilent Technologies). The valves involved are a nanovolume injection valve (C74MPKH-467, VICI) and a manual 8-port switching valve with 1/16 ports (C2H-1000, VICI) to divert the mobile phase delivery from the microchip. In-line pressure sensors (Duratec, Germany) are integrated to measure the pre- and post-column pressure.

### An overview of all capillaries involved is listed in the following. (a) – PEEK, OD 1/16, ID 180 µm, 25 cm, (b + c) – restriction capillaries (Agilent G1312-67500), (d) – PEEK, OD 1/16, ID 100 µm, 30 cm and OD 360 µm, ID 100µm, 30cm, (e) - PEEK, OD 360 µm, ID 125 µm, 10 cm, (f) – PEEK, OD 360 µm, ID 125 µm, 15 cm, (g) – PEEK, OD 360 µm, ID 50 µm, 15 cm, (h) – PEEK, OD 360 µm, ID 100 µm, 10 cm, sealed by PEEK cap, (i) – PEEK, OD 360 µm, ID 50 µm, 15 cm, (j) PEEK, OD 360 µm, ID 100 µm, 15 cm, (k) PEEK, OD 360 µm, ID 50 µm, 15 cm and SST, OD 1/16, ID 120 µm, 12.5 cm, (l) – SST, OD 1/16, ID 200 µm, 120 cm + heater cartridge, (m) – PEEK, OD 360 µm, ID 100 µm, 15 cm, sealed by PEEK cap, (n) – PEEK, OD 360 µm, ID 100 µm, 30 cm, (o) – PEEK, OD 360 µm, ID 100,µm, 15 cm, (p) – PEEK, OD 360 µm, ID 100 µm, 30 cm and PEEK, OD 1/16, ID 120 µm, 10 cm, (q) – PEEK, OD 1/16 ID 120 µm, 10 cm, (BPR unit 1) – static 1000 psi and dynamic backpressure regulator (1300-4200 psi) connected via SST OD 1/16 ID 120 µm 12.5 cm, (BPR unit 2) – static 1000psi and dynamic backpressure regulator (1300-4200 psi) connected via SST OD 1/16 ID 120µm 12.5cm, both BPR are heated and submerged in IPA to prevent damage caused by the CO_2_-based eluent.

### The optical setup for on-chip fluorescence detection was integrated into an inverted microscope (IX 71, Zeiss, Germany) and consisted of a (1) 40x magnification lens (LUCPlanFLN, Olympus), (2) filter cube equipped with an excitation bandpass filter 350/50nm, dichroic mirror 380nm and an emission long-pass filter 390nm, (3) Photomultiplier tube (H9305-03, Hamamatsu) and (4) a mercury vapor lamp as UV-source equipped with a Osram HBO 101W.

## Figure S2 –Impact of mobile phase modifier

Since supercritical CO_2_ (scCO_2_) has weak eluting strength, methanol is added to the mobile phase to enhance retention times by modifying the solvation strength. Given that low concentrations of the selected analytes are soluble in methanol, it was anticipated that a small amount of this modifier would improve retention. This effect was demonstrated during optimizing the mobile phase for chipSFC separation, as depicted in Fig. S2. Here, increasing the methanol content to 20% resulted in the fastest retention times. Higher methanol content resulted in longer retention due to reduced analyte solubility in the more polar mobile phase, reflecting competitive adsorption dynamics between the mobile phase and analyte.


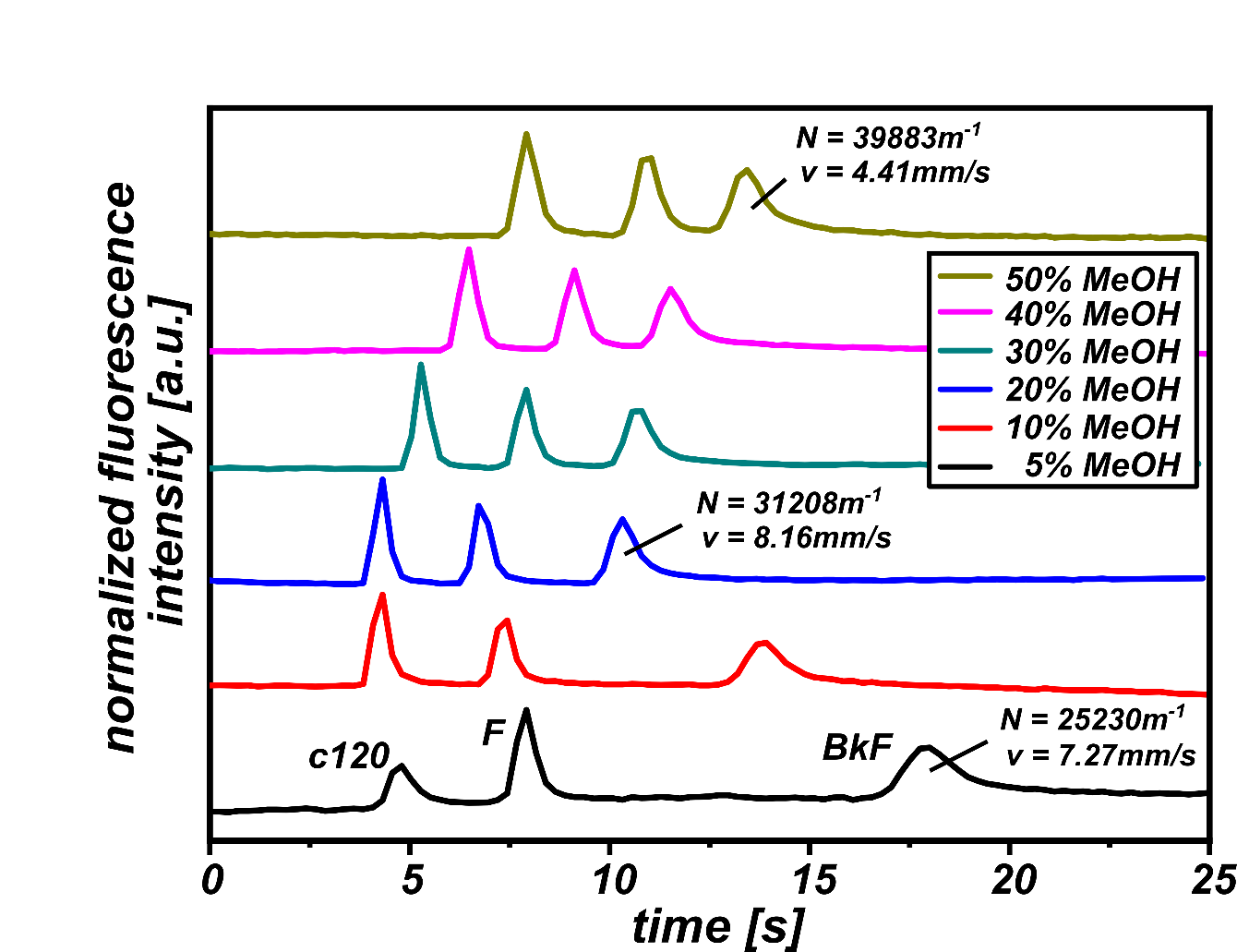


### **Fig. S2** Impact of mobile phase modifier content illustrated by normalized chipSFC chromatograms acquired under modifier proportions ranging from 5 to 50% v/v methanol. Linear velocities are calculated based on retention of c120. Theoretical plates are based on BkF. Pre-column pressure: 160 bar, split injection mode, post-column pressure: 120bar, stationary phase: C18 BEH, dp=2,5µm, column length: 35mm, temperature: 60 °C, eluent: CO_2_/MeOH 80:20 (v/v), fluorescence detection, sample mixture dissolved in MeOH: 7-amino-4-methylcoumarine (C120): 400 μmol/L, Fluoranthene (F): 1.90 mmol/L, Benzo[k]fluoranthene (BkF): 200 μmol/L.

## Figure S3 – Impact of column pressure drop

Due to the low viscosity of the CO_2_/methanol mobile phase in supercritical fluid chromatography (SFC), there is typically a low-pressure drop across the column. Post-column pressure was adjusted to circumvent the low-pressure drops and provoke higher velocities across the column. Since fluorescence detection takes place under high-pressure conditions, a conventional backpressure regulator can be used for this.

The impact of varying pressure drops (ranging from 30 to 80 bar) on chromatography performance is illustrated in Fig. S3. Inhere, increasing pressure drops resulted in higher flow velocities (from 6.06 up to 12.98 mm/s), but the relationship is non-linear and flattens with increased pressure drops. Higher pressure drops also result in reduced post-column pressures, which can potentially cause phase separation and analyte precipitation. Therefore, a post-column pressure of 120 bar (or higher) was selected for further measurements to provide a moderate pressure drop (40 bar) for efficient separation performance while ensuring solubility and homogeneity of the effluent.

**
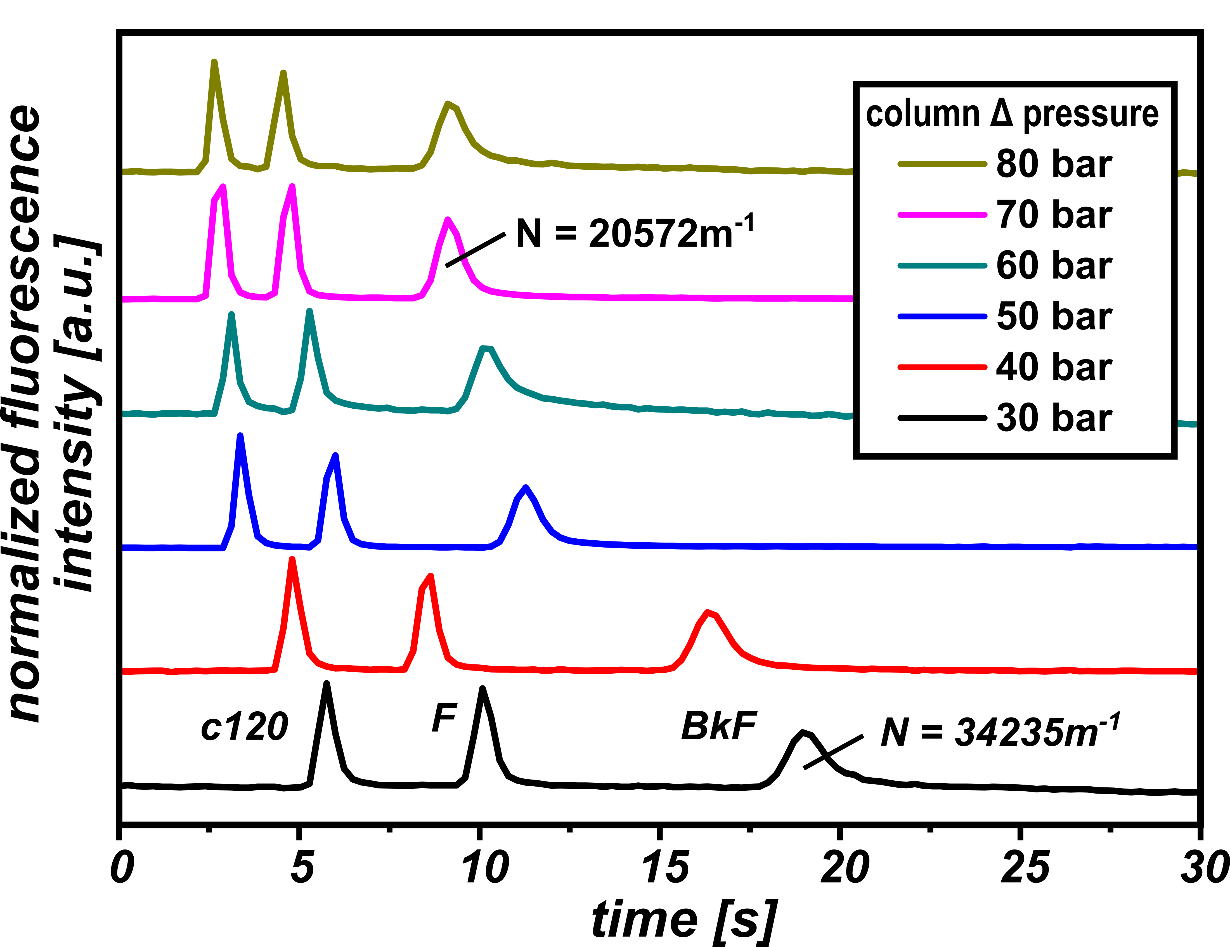
**

### **Fig. S3** Impact of pressure drop illustrated by normalized chipSFC chromatograms acquired under post-column pressures ranging from 80 to 130bar. Linear velocities are calculated based on retention of c120. Theoretical plates are based on BkF. Pre-column pressure: 160 bar, split injection mode, stationary phase: C18 BEH, dp=2,5µm, column length: 35mm, temperature: 60 °C, eluent: CO_2_/MeOH 80:20 (v/v), fluorescence detection, sample mixture dissolved in MeOH: 7-amino-4-methylcoumarine (C120): 400 μmol/L, Fluoranthene (F): 1.90 mmol/L, Benzo[k]fluoranthene (BkF): 200 μmol/L.

##
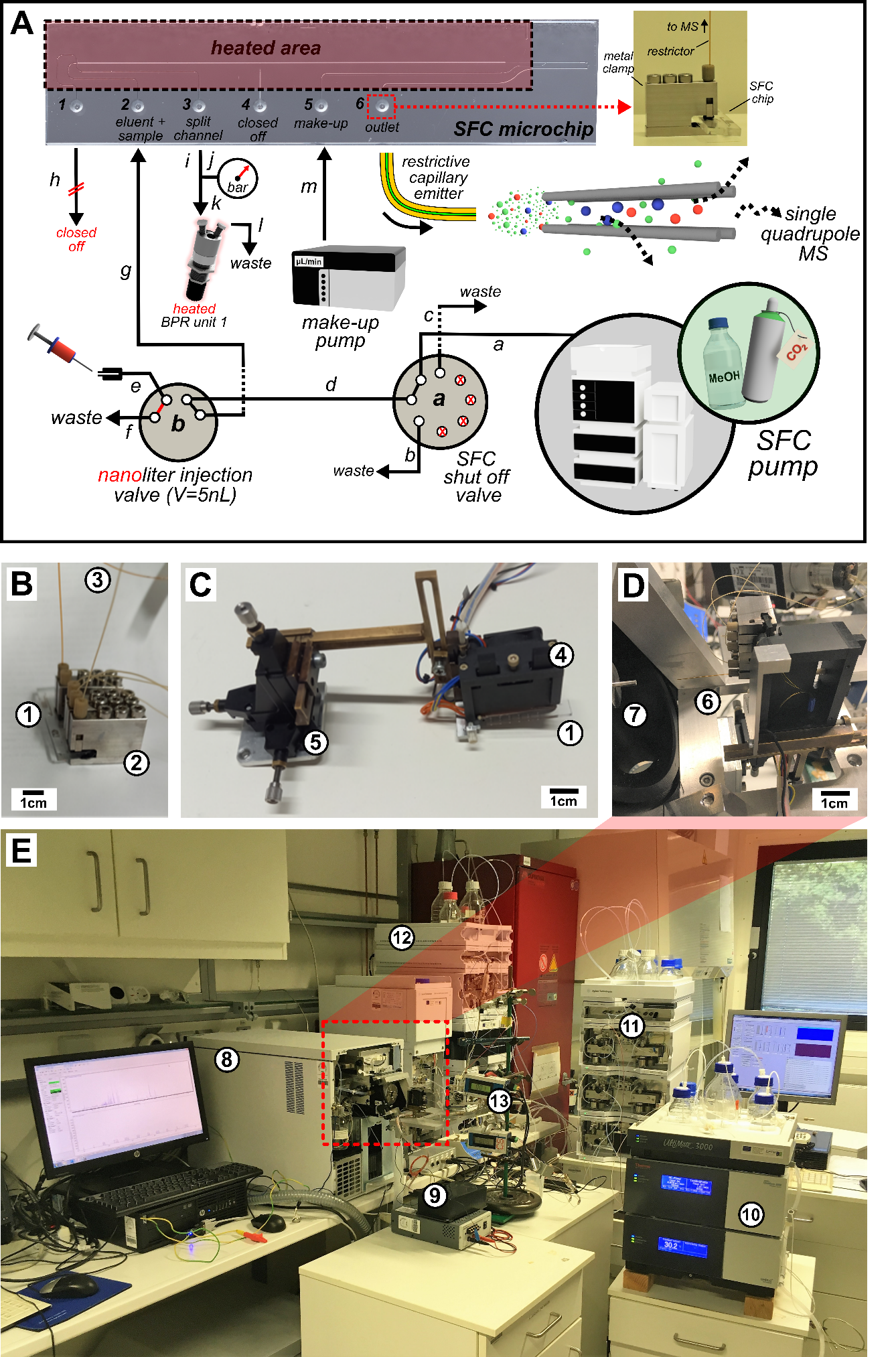
Figure S4 – chipSFC MS setup

### **Fig. S4** Overview of the chipSFC MS setup. (**A**) Schematic illustration of the instrumental setup used for chipSFC MS in this study. The majority of the fluidic circuitry is consistent with that used during fluorescence measurements, with specific differences highlighted here. The strategies employed for backpressure regulation and detector coupling are main differences compared to the fluorescence setup. Backpressure regulation utilizes a make-up stream. Therefore, port 5, previously closed off, was reopened to allow make-up dosing delivered via capillary connection (m – PEEK, OD 1/16, ID130μm, 40cm and PEEK, OD 360μm, ID100μm, 25cm) using a nano pump (Ultimate NCS-3500RS, Thermo Fischer Scientific, USA) or HPLC pump (1260 Infinity, Agilent Technologies, USA). The pressure values recorded by the make-up pump were used to track the make-up pressure. Detector coupling was achieved using a fused silica capillary. Consequently, the backpressure regulator unit (BPR unit 2 including capillaries n, o, p, and q, along with a pressure sensor as seen in Fig. S1) in the post-column section was disconnected from the outlet of the SFC microchip. The fused silica capillary was connected to the outlet port of the SFC microchip using metal clamps. This capillary serves as an emitter for MS detection and contributes to post-column flow restriction. Single quadrupole mass spectrometry was utilized for mass analysis.

### Photographic images of the instrumental setup are shown, including (1) SFC microchip with (2) homemade metal clamps for (3) peripheral fluid connections shown in Fig. S4 B. (4) Chip thermostat and (5) XYZ-micromanipulator included in the microchip assembly in Fig. S4 C. (6) chipSFC equipped with a restrictive capillary emitter in front of the (7) MS inlet in Fig. S4 D. The entire instrumental setup including (8) Single Quadrupole MS, (9) chip thermostat electronics, (10) nanoflow pump, (11) HPLC pump, (12) SFC pump, and (13) pressure sensors and valving in Fig. S4 E.

## Figure S5 - Evaluation of different restrictor dimensions

In make-up-assisted backpressure regulation, pressure control hinges on the interplay between a viscous make-up fluid and a restrictive post-column environment. In the developed chipSFC configuration, this environment is characterized by a fused silica capillary restrictor connected at the microchip outlet. To investigate this interaction, we examined different dimensions of the restrictor concerning the make-up flow rate to determine their influence on the achievable range of backpressures.

The backpressure data corresponding to the three different restrictor dimensions are depicted in Fig. S5. Analysis of the results revealed three distinct trends, which can be elucidated using the Hagen-Poiseuille Equation. Firstly, higher viscosity of the make-up fluid necessitates lower flow rates to achieve a specific pressure compared to lower-viscosity compositions. Secondly, increasing the flow rate results in higher backpressures. Lastly, larger restrictor dimensions demand higher make-up flow rates to reach a desired pressure, albeit enabling coverage of a wider pressure range. Based on the results of this preliminary experiment, a capillary restrictor with ID 20μm and a length of 80cm was selected for the chipSFC MS experiments.


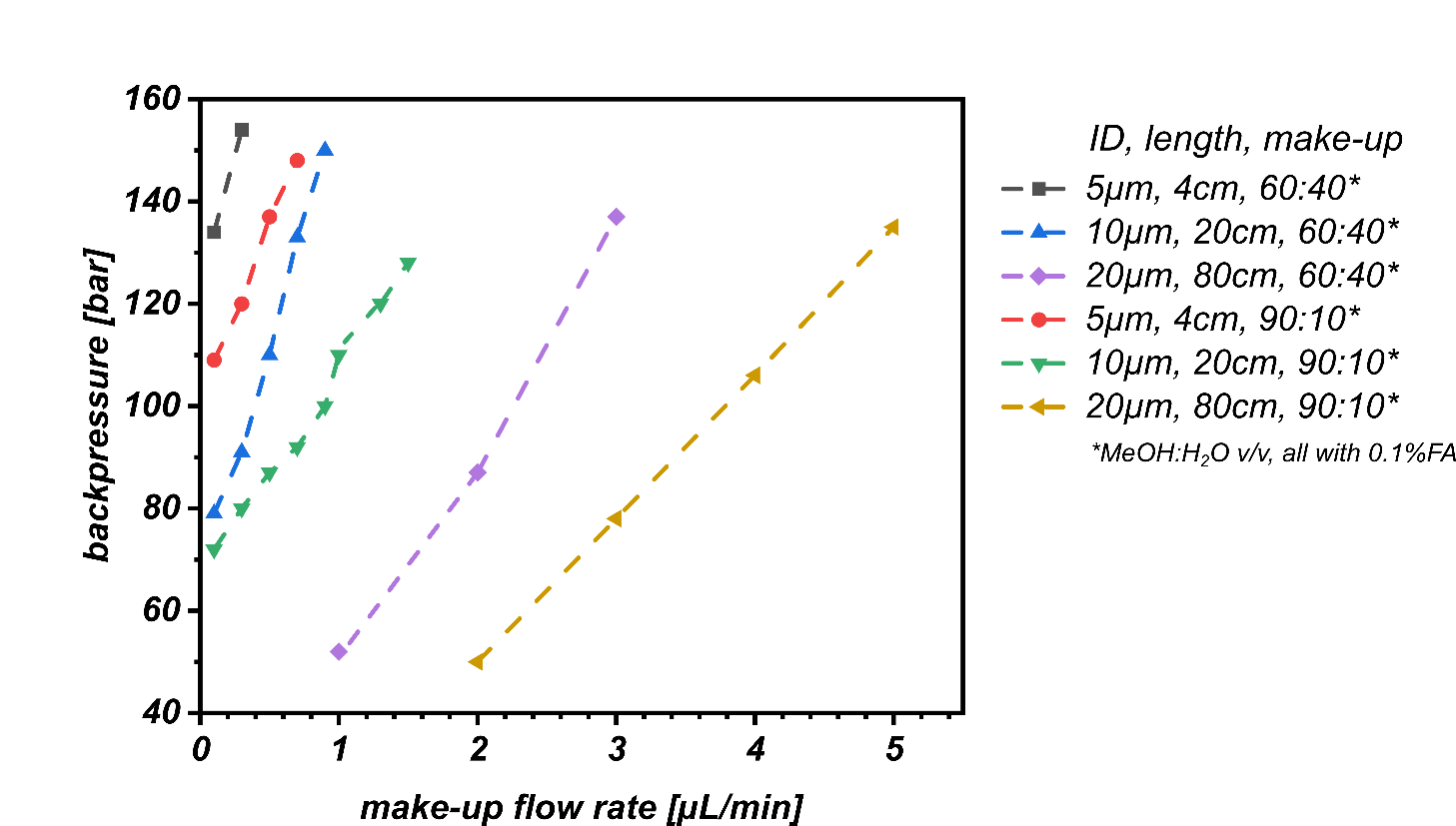


### **Fig. S5** Backpressure of fused silica capillary as a function of make-up flow rates. Fused silica capillaries of three different dimensions, (1) ID 5µm, length 4.5cm, (2) ID 10µm, length 20cm and (3) ID20µm and length 80cm were evaluated for make-up flow rates from 0.1 to 5µL/min using two make-up fluid compositions (90:10 v/v Methanol:H_2_O or 60:40 v/v Methanol:H_2_O, both with 0.1%FA) for an eluent of 80:20 (v/v) CO_2_/MeOH at 160 bar

##
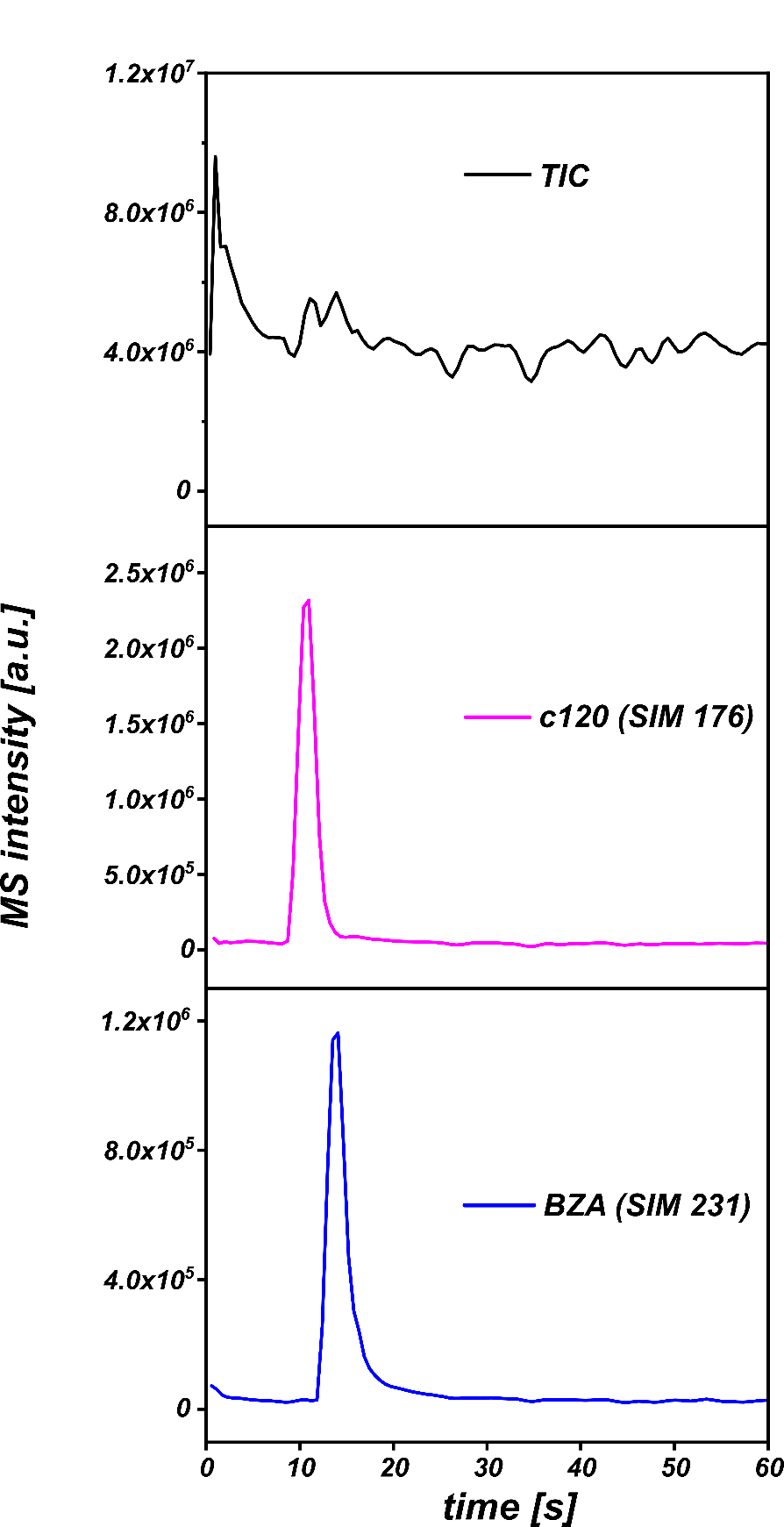
Figure S6 – Ion chromatograms acquired by chipSFC MS

### **Fig. S6** TIC and SIM chromatograms for m/z 176 (c120) and m/z 231 (BZA) acquired by chipSFC MS. MS parameter are listed in the following: positive ion mode, mass range of 150-400m/z for TIC, capillary voltage -4kV, dry gas flow rate 3l/min, dry gas temperature 350°C, fragmentor voltages 200V for c120, 230V for BZA, sample mixture: c120 and BZA each 1mmol/L, chromatographic parameter listed below, stationary phase: C18 BEH, dp=2.5µm, column length 35mm, pre-column pressure: 160bar, post-column pressure:130bar, eluent: 80:20 v/v CO_2_:MeOH, make-up: 5µL/min of a 90:10 v/v MeOH:H_2_O, 0.1%FA, restrictor dimensions, capillary length 80cm, inner diameter 20µm

## Figure S7 – Impact of temperature

Due to the presence of the microchip thermostat, the chipSFC microchip was capable of being heated, which, in combination with the use of a temperature-stable stationary phase (BEH C18), allowed us to maintain the mobile phase in the supercritical state.

In Fig. S7 the model mixture was separated at different temperatures, demonstrating a shift in retention times following an increase in temperature. This shift is attributed to the density changes of the mobile phase (80:20 v/v CO_2_:MeOH) as it transitions from subcritical to supercritical conditions at the column inlet when the temperature is raised from 20°C to 90°C [4]. As the mobile phase migrates through the column, these density variations diminish because post-column pressure was kept constant for both separations.

By using a restrictive capillary emitter (ID 20μm, length 80cm), most of the post-column pressure drop occurs off-chip, where temperature control is not applied. The rapid heat exchange in smaller dimensions suggests that the processes within the capillary emitter occur at room temperature.


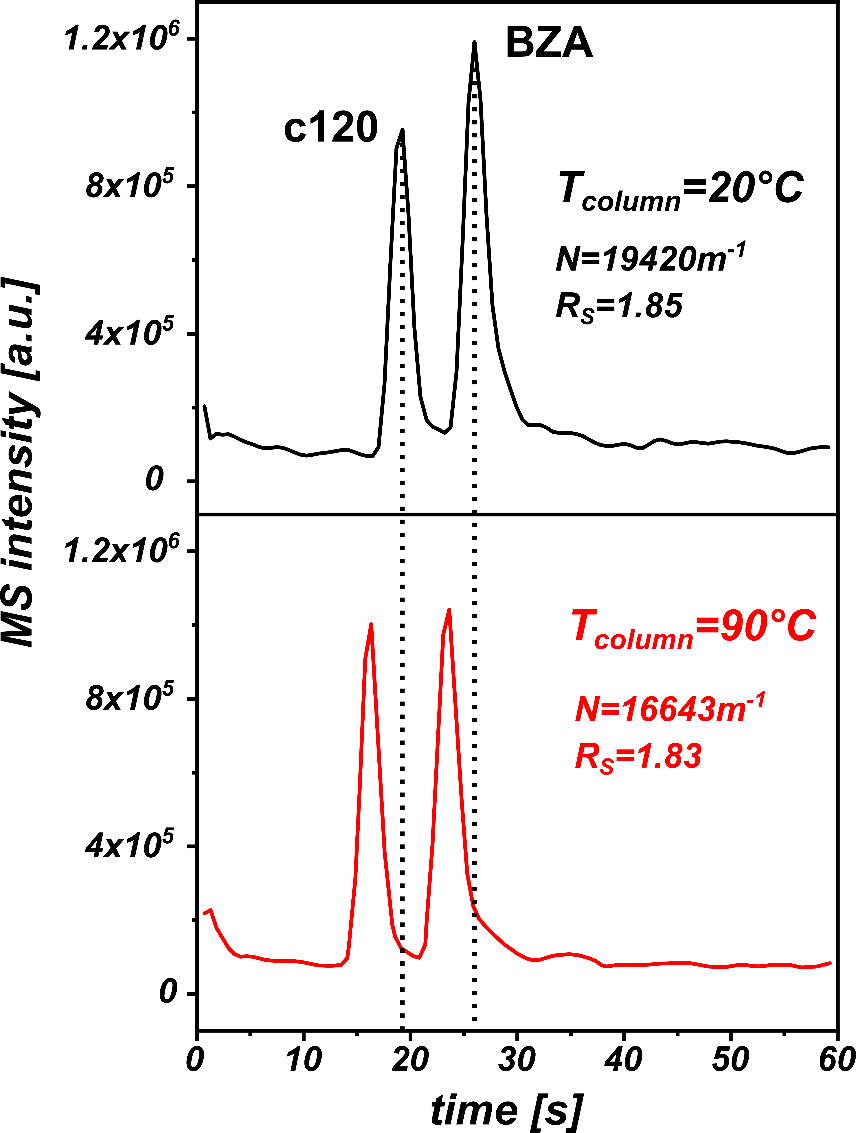


### **Fig. S7** Impact of temperature on the separation of model mixture by chipSFC MS, chromatographic parameter listed below, split-injection mode, stationary phase: C18 BEH, dp=2.5µm, column length 35mm, pre-column pressure: 140bar, post-column pressure:123bar, eluent: 80:20 v/v CO_2_:MeOH, make-up: 5µL/min of a 90:10 v/v MeOH:H_2_O, 0.1%FA, restrictor dimensions, capillary length 80cm, inner diameter 20µm, MS parameter are listed in the following: positive ion mode, BPC of m/z 176 and m/z 231, cycle time 500ms, capillary voltage -4kV, dry gas flow rate 3l/min, dry gas temperature 350°C, fragmentor voltages 200V for c120, 230V for BZA, sample mixture: c120 and BZA each 1mmol/L

##
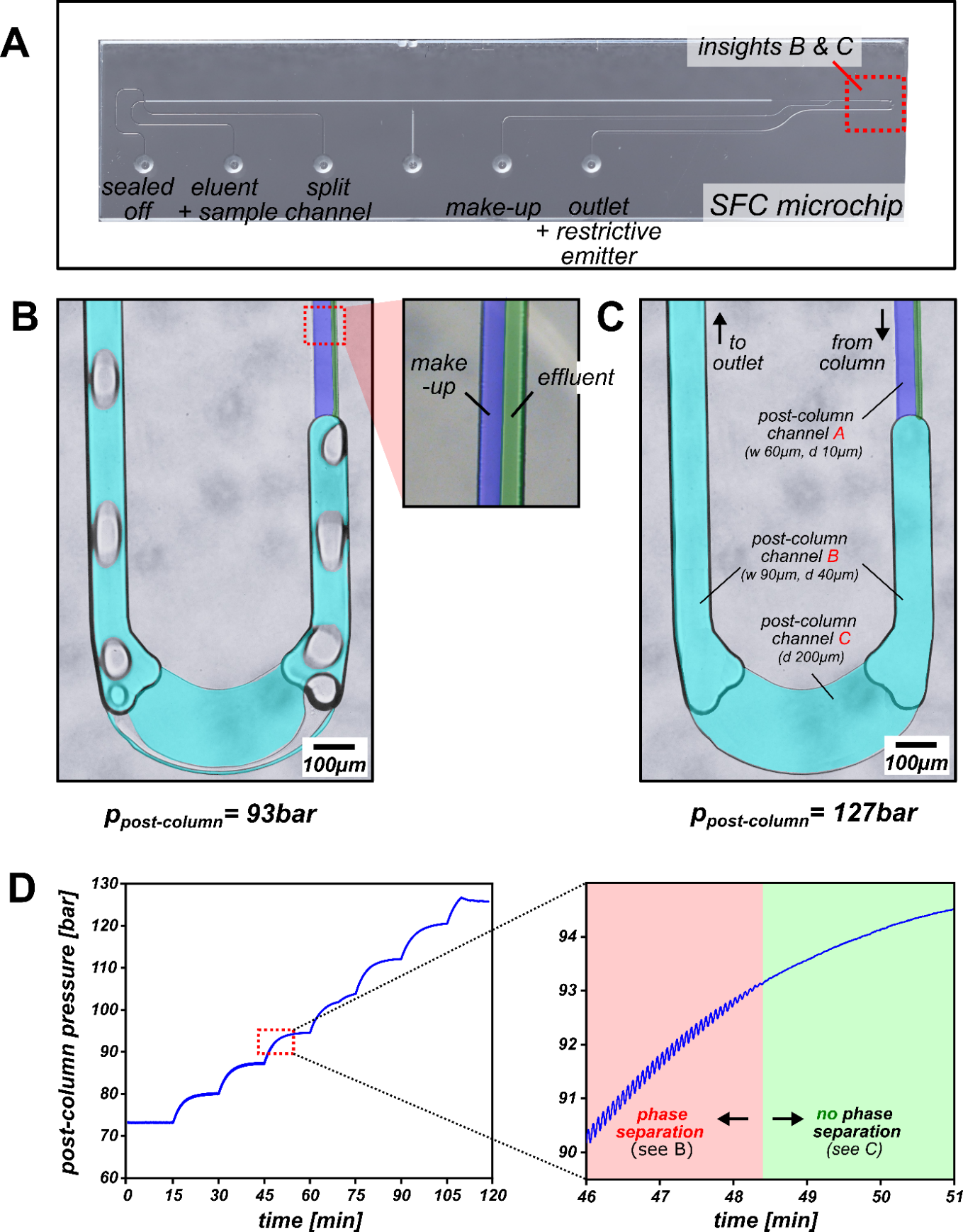
Figure S8 – Observation in the post-column section

### **Fig. S8** Observation in the post-column section of the SFC microchip. (**A**) Location of the observed post-column section on the SFC microchip. Photographic insights of the enlarged post-column channel section at different post-column pressures: (**B**) 93 bar, (**C**) 127 bar. (**D**) Post-column pressure curve obtained during make-up assisted backpressure regulation. The insight shows the transition from a region with significant pressure ripple to no pressure ripple caused by the successive absence of the phase separation. Experimental parameters are listed below eluent: 80:20 v/v CO_2_:MeOH, post-column pressure: 93bar, make-up conditions: 2µL/min of 60:40 v/v MeOH:H_2_O, 0.1% eluent, restrictor: length 80cm, ID 20µm, w stands for channel width, d stands for channel depth

The results in Fig. S8 highlight the importance of selecting appropriate channel dimensions. The post-column section of the SFC microchip comprises channels with trapezoidal cross-sections of various dimensions (Fig. S8A). Operating these channel sections under low-pressure conditions (below 93 bar) results in phase separation caused by the rapid pressure drop due to channel enlargement, as seen in Fig. S8B. Make-up-assisted pressure regulation prevents this phase separation (Fig. S8C) and induces a highly stable post-column pressure without pressure ripple (Fig. S8D). However, the developed pressure regulation cannot prevent mixing between the effluent and make-up flow, indicating the need to adapt channel dimensions in future chip designs.

References

1. Thurmann S, Dittmar A, Belder D. A low pressure on-chip injection strategy for high-performance chip-based chromatography. J Chromatogr A. 2014; https://doi.org/10.1016/j.chroma.2014.03.009

2. Thurmann S, Mauritz L, Heck C, Belder D. High-performance liquid chromatography on glass chips using precisely defined porous polymer monoliths as particle retaining elements. J Chromatogr A. 2014; https://doi.org/10.1016/j.chroma.2014.10.008

3. Heiland JJ, Lotter C, Stein V, Mauritz L, Belder D. Temperature Gradient Elution and Superheated Eluents in Chip-HPLC. Anal Chem. 2017; https://doi.org/10.1021/acs.analchem.7b00142

4. Saito M. History of supercritical fluid chromatography: instrumental development. J Biosci Bioeng. 2013; https://doi.org/10.1016/j.jbiosc.2012.12.008
